# Supplementary material for: Crop classification with UAV multispectral remote sensing, employing an enhanced ResNet50 residual network
Source: PLoS One. 2025 Dec 30;20(12):e0339644. doi: 10.1371/journal.pone.0339644 (PMC12752950; doi:10.1371/journal.pone.0339644)
Supplement: Attachment — Submitted filename: Report on PONE-D-25-38216.pdf [file pone.0339644.s001.pdf]

Report on the manuscript:

Title: An Investigation into Crop Classification with UAV Multispectral Remote Sensing, Employing an Enhanced ResNet50 Residual Network.

Manuscript number: PONE-D-25-38216

This paper investigates the use of UAV multispectral imagery combined with an enhanced ResNet50 deep residual network to improve crop classification, focusing on flue-cured tobacco and maize. The authors conduct ablation experiments to identify the optimal spectral band combination (RGB+NIR+Edge) and propose architectural improvements to ResNet50, including the integration of Batch Normalization (BN) layers, pyramid pooling layers (PPL), and hidden layers. They systematically evaluate seven enhancement strategies and find that the inclusion of BN layers alone yields the most significant performance gain, achieving an accuracy of 94.90% and an F1 score of 94.93%. The study concludes that combining NIR and edge bands significantly boosts classification accuracy and that BN layers provide an optimal trade-off between performance and model complexity.

According to best of my knowledge, the authors addresses the need for precise crop classification in precision agriculture using UAV multispectral data includes both ablation studies and model improvement experiments to assess the impact of architectural changes. The inclusion of multiple evaluation metrics such as, accuracy, precision, recall, kappa coefficient, F1 score and confusion matrix visualization strengthens the credibility of the findings. These finding offers a practical pipeline for small-scale agricultural monitoring and demonstrates that enhanced ResNet50 can outperform the baseline model with minimal complexity overhead.

Recommendation:

The presented work in this manuscript is technically vigorous and well-structured, with strong experimental validation, the paper is suitable for publication. Therefore, I **recommend** this manuscript for publication in PLOS ONE.
