# Supplementary material for: Crop classification with UAV multispectral remote sensing, employing an enhanced ResNet50 residual network
Source: PLoS One. 2025 Dec 30;20(12):e0339644. doi: 10.1371/journal.pone.0339644 (PMC12752950; doi:10.1371/journal.pone.0339644)
Supplement: Attachment — Submitted filename: Response letter.docx [file pone.0339644.s002.docx]

**Response to Editorial and Referee Comments**

We sincerely thank the editors and reviewers for their valuable suggestions, which we have incorporated into the comprehensive revision of the manuscript. In accordance with the reviewers' requests, we elaborated on the mechanism of multispectral band complementarity, thoroughly demonstrated the effectiveness of various ResNet50 enhancement approaches, and conducted an in-depth analysis of the impact of different augmentation modules on the model generalization robustness (F1-sd) and training stability. Below are our point-by-point responses to each reviewer's comment.

1. Please ensure that your manuscript meets PLOS ONE's style requirements, including those for file naming. The PLOS ONE style templates can be found at https://journals.plos.org/plosone/s/file?id=wjVg/PLOSOne_formatting_sample_main_body.pdf and https://journals.plos.org/plosone/s/file?id=ba62/PLOSOne_formatting_sample_title_authors_affiliations.pdf

Answer：Thank you for your reminder. We would like to confirm that we have carefully reviewed the style templates provided by your journal and have revised our manuscript accordingly. The manuscript now fully adheres to all formatting requirements of PLOS ONE, including the file naming conventions.

2.Please note that PLOS One has specific guidelines on code sharing for submissions in which author-generated code underpins the findings in the manuscript. In these cases, we expect all author-generated code to be made available without restrictions upon publication of the work. Please review our guidelines at https://journals.plos.org/plosone/s/materials-and-software-sharing#loc-sharing-code and ensure that your code is shared in a way that follows best practice and facilitates reproducibility and reuse.

Answer：Thank you for your reminder regarding code sharing. We hereby confirm full compliance with PLOS ONE guidelines:

Code repository and access: All code supporting this study's findings, generated by the authors, has been permanently stored and made publicly accessible in Zenodo, a recognized universal open knowledge repository, with unrestricted access. Persistent identifier: The code is accessible via the following permanent DOI link: https://doi.org/10.5281/zenodo.17451761

Reuse License: As indicated in the “Rights” section of the Zenodo repository page, the code is released under the MIT License. This license explicitly permits unrestricted use, distribution, and modification of the code by anyone, provided the original copyright notice is retained. This fully complies with your journal's “unrestricted use” policy requirement.

3.We note that you have indicated that there are restrictions to data sharing for this study. PLOS only allows data to be available upon request if there are legal or ethical restrictions on sharing data publicly. For more information on unacceptable data access restrictions, please see <http://journals.plos.org/plosone/s/data-availability#loc-unacceptable-data-access-restrictions.>

4.In the online submission form, you indicated that the data that support the findings of this study are available on request from the corresponding author. The data are not publicly available due to privacy or ethical restrictions.

Answer：Thank you for the editor's reminder. Regarding data availability, we hereby clarify that the relevant data for this study is now publicly available without any restrictions in the Zenodo repository. The data access link (DOI) is: https://doi.org/10.5281/zenodo.17451761.We have accordingly updated the DOI citation for this public dataset in the “Data Availability Statement” section of the manuscript and removed the previous reference to “available upon request.” We sincerely apologize for any confusion caused by the initially inaccurate option selected in the submission form. The current data sharing approach is now fully compliant with PLOS ONE's data disclosure policy.

5. Thank you for stating the following financial disclosure:
This work was supported by the National Key R & D Program (2024YFD1700104).

Please state what role the funders took in the study. If the funders had no role, please state: "The funders had no role in study design, data collection and analysis, decision to publish, or preparation of the manuscript."
If this statement is not correct you must amend it as needed.
Please include this amended Role of Funder statement in your cover letter; we will change the online submission form on your behalf.

Answer：The funders had no role in study design, data collection and analysis, decision to publish, or preparation of the manuscript.

6. Thank you for stating the following in the Acknowledgments Section of your manuscript:
First and foremost, I extend my heartfelt gratitude to my tutor, Professor Jianxiong Wang, whose invaluable guidance and financial support were crucial to the completion of this thesis. Additionally, I deeply appreciate my classmate Shixian Lu and my two close friends, who offered insightful suggestions and hands-on assistance during the experimental and writing phases. This work was supported by the National Key R & D Program (2024YFD1700104).

We note that you have provided funding information that is not currently declared in your Funding Statement. However, funding information should not appear in the Acknowledgments section or other areas of your manuscript. We will only publish funding information present in the Funding Statement section of the online submission form.
Please remove any funding-related text from the manuscript and let us know how you would like to update your Funding Statement. Currently, your Funding Statement reads as follows:
This work was supported by the National Key R & D Program (2024YFD1700104).

Please include your amended statements within your cover letter; we will change the online submission form on your behalf.

Answer：Thank you for your reminder. We have fully removed all fund-related text from the “Acknowledgments” section of the manuscript as requested. Regarding the funding statement, we confirm no further updates are necessary. The existing statement in the online submission system—“This work was supported by the National Key R&D Program (2024YFD1700104)”—is the complete and correct final version. Please consider this as the definitive version. Thank you!

7. Please amend your list of authors on the manuscript to ensure that each author is linked to an affiliation. Authors’ affiliations should reflect the institution where the work was done (if authors moved subsequently, you can also list the new affiliation stating “current affiliation:….” as necessary).

Dear Editor,

Thank you for your prompt attention. Regarding the author list and affiliation information in our manuscript, we encountered technical limitations during online submission. We kindly request your assistance in updating the following details in the backend:

Affiliation Corrections:

As the system database could not retrieve the full institutional names for the following authors, please manually update their affiliations:

Chenwei Xu's affiliation should be: Yunnan Agricultural University, Yunnan Provincial International Joint Research and Development Center for Smart Environment, Yunnan Agricultural University, Kunming 605201, China.

Shixian Lu's affiliation should be: Yunnan Agricultural University, Yunnan Provincial International Joint Research and Development Center for Smart Environment, Yunnan Agricultural University, Kunming 605201, China.

Xiang Feng's affiliation should be: Agricultural Economics and Informatics Research Institute,Yunnan Academy of Agricultural Sciences.

Xuelin Zhang's affiliation should be: Beijing Century Guoyuan Technology Co., Ltd., Beijing, 100032, China.

Jianxiong Wang should be affiliated with Yunnan Agricultural University, Yunnan Provincial International Joint Research and Development Center for Smart Environment, Yunnan Agricultural University, Kunming 605201, China.

Corresponding author designation: Professor Jianxiong Wang should be designated as the corresponding author for this paper. His email address is: wangjx@ynau.edu.cn. As the system cannot automatically associate his account with the manuscript during retrieval, we were unable to designate him as the corresponding author during submission. We kindly request that you manually add him and activate his corresponding author permissions in the system backend. We sincerely apologize for any inconvenience caused and deeply appreciate your understanding and valuable assistance.

8. [We note that Figure(s) 1, 2 and 6, in your submission contain [map/satellite] images which may be copyrighted. All PLOS content is published under the Creative Commons Attribution License (CC BY 4.0), which means that the manuscript, images, and Supporting Information files will be freely available online, and any third party is permitted to access, download, copy, distribute, and use these materials in any way, even commercially, with proper attribution. For these reasons, we cannot publish previously copyrighted maps or satellite images created using proprietary data, such as Google software (Google Maps, Street View, and Earth). For more information, see our copyright guidelines: http://journals.plos.org/plosone/s/licenses-and-copyright.

   We require you to either (1) present written permission from the copyright holder to publish these figures specifically under the CC BY 4.0 license, or (2) remove the figures from your submission:

   a. You may seek permission from the original copyright holder of Figure(s) 1, 2 and 6, to publish the content specifically under the CC BY 4.0 license.

   We recommend that you contact the original copyright holder with the Content Permission Form (http://journals.plos.org/plosone/s/file?id=7c09/content-permission-form.pdf) and the following text:
   “I request permission for the open-access journal PLOS ONE to publish XXX under the Creative Commons Attribution License (CCAL) CC BY 4.0 (http://creativecommons.org/licenses/by/4.0/). Please be aware that this license allows unrestricted use and distribution, even commercially, by third parties. Please reply and provide explicit written permission to publish XXX under a CC BY license and complete the attached form.”

   Please upload the completed Content Permission Form or other proof of granted permissions as an "Other" file with your submission.

   In the figure caption of the copyrighted figure, please include the following text: “Reprinted from [ref] under a CC BY license, with permission from [name of publisher], original copyright [original copyright year].”

   b. If you are unable to obtain permission from the original copyright holder to publish these figures under the CC BY 4.0 license or if the copyright holder’s requirements are incompatible with the CC BY 4.0 license, please either i) remove the figure or ii) supply a replacement figure that complies with the CC BY 4.0 license. Please check copyright information on all replacement figures and update the figure caption with source information. If applicable, please specify in the figure caption text when a figure is similar but not identical to the original image and is therefore for illustrative purposes only.
   The following resources for replacing copyrighted map figures may be helpful:

   USGS National Map Viewer (public domain): http://viewer.nationalmap.gov/viewer/
   The Gateway to Astronaut Photography of Earth (public domain): http://eol.jsc.nasa.gov/sseop/clickmap/
   Maps at the CIA (public domain): https://www.cia.gov/library/publications/the-world-factbook/index.html and https://www.cia.gov/library/publications/cia-maps-publications/index.html
   NASA Earth Observatory (public domain): http://earthobservatory.nasa.gov/
   Landsat: http://landsat.visibleearth.nasa.gov/
   USGS EROS (Earth Resources Observatory and Science (EROS) Center) (public domain): http://eros.usgs.gov/#
   Natural Earth (public domain): http://www.naturalearthdata.com/](http://track.editorialmanager.com/CL0/We%20note%20that%20Figure(s)%201,%202%20and%206,%20in%20your%20submission%20contain%20%5bmap/satellite%5d%20images%20which%20may%20be%20copyrighted.%20All%20PLOS%20content%20is%20published%20under%20the%20Creative%20Commons%20Attribution%20License%20(CC%20BY%204.0),%20which%20means%20that%20the%20manuscript,%20images,%20and%20Supporting%20Information%20files%20will%20be%20freely%20available%20online,%20and%20any%20third%20party%20is%20permitted%20to%20access,%20download,%20copy,%20distribute,%20and%20use%20these%20materials%20in%20any%20way,%20even%20commercially,%20with%20proper%20attribution.%20For%20these%20reasons,%20we%20cannot%20publish%20previously%20copyrighted%20maps%20or%20satellite%20images%20created%20using%20proprietary%20data,%20such%20as%20Google%20software%20(Google%20Maps,%20Street%20View,%20and%20Earth).%20For%20more%20information,%20see%20our%20copyright%20guidelines:%20http:/journals.plos.org/plosone/s/licenses-and-copyright.%0b%0bWe%20require%20you%20to%20either%20(1)%20present%20written%20permission%20from%20the%20copyright%20holder%20to%20publish%20these%20figures%20specifically%20under%20the%20CC%20BY%204.0%20license,%20or%20(2)%20remove%20the%20figures%20from%20your%20submission:%0b%0ba.%20You%20may%20seek%20permission%20from%20the%20original%20copyright%20holder%20of%20Figure(s)%201,%202%20and%206,%20to%20publish%20the%20content%20specifically%20under%20the%20CC%20BY%204.0%20license.%20%0b%0bWe%20recommend%20that%20you%20contact%20the%20original%20copyright%20holder%20with%20the%20Content%20Permission%20Form%20(http:/journals.plos.org/plosone/s/file?id=7c09/content-permission-form.pdf)%20and%20the%20following%20text:%0b%E2%80%9CI%20request%20permission%20for%20the%20open-access%20journal%20PLOS%20ONE%20to%20publish%20XXX%20under%20the%20Creative%20Commons%20Attribution%20License%20(CCAL)%20CC%20BY%204.0%20(http://creativecommons.org/licenses/by/4.0/).%20Please%20be%20aware%20that%20this%20license%20allows%20unrestricted%20use%20and%20distribution,%20even%20commercially,%20by%20third%20parties.%20Please%20reply%20and%20provide%20explicit%20written%20permission%20to%20publish%20XXX%20under%20a%20CC%20BY%20license%20and%20complete%20the%20attached%20form.%E2%80%9D%0b%0bPlease%20upload%20the%20completed%20Content%20Permission%20Form%20or%20other%20proof%20of%20granted%20permissions%20as%20an%20%22Other%22%20file%20with%20your%20submission.%0b%0bIn%20the%20figure%20caption%20of%20the%20copyrighted%20figure,%20please%20include%20the%20following%20text:%20%E2%80%9CReprinted%20from%20%5bref%5d%20under%20a%20CC%20BY%20license,%20with%20permission%20from%20%5bname%20of%20publisher%5d,%20original%20copyright%20%5boriginal%20copyright%20year%5d.%E2%80%9D%0b%0bb.%20If%20you%20ar/1/010f01997eea5760-ac3e560a-6da8-4a38-a8e6-de06eea7c05f-000000/NwnxG_sObw0OtghUJnEsVXhVzFXFgudnC7i8FjgR0lk=230)

Answer：Figure(s) 1, 2 . Satellite image of the study area.

Base image: Sentinel-2 Level-2A surface reflectance (10 m), tile T50RQQ, acquisition date 2023-09-14. This product contains modified Copernicus Sentinel data [2023], processed by the European Space Agency (ESA). The use of Sentinel data is governed by the Copernicus Programme's free and open data policy, which permits unrestricted use, redistribution, and creation of derivative works, including for commercial purposes (https://sentinels.copernicus.eu/documents/247904/690755/Sentinel_Data_Legal_Notice). This license is compatible with the Creative Commons Attribution (CC BY) 4.0 license.The image was processed and exported in Google Earth Engine (Gorelick et al. 2017) to a 32-bit GeoTIFF, reprojected to WGS 84 / UTM zone 50N, and clipped to the study area.

Figure 8. UAV imagery from Prediction Zone.Data availability: The UAV imagery presented in this figure is the original work of the authors. The underlying image data are available as part of the complete dataset in the Zenodo repository: [https://doi.org/10.5281/zenodo.17451761](https://doi.org/10.5281/zenodo.17451761).

9. If the reviewer comments include a recommendation to cite specific previously published works, please review and evaluate these publications to determine whether they are relevant and should be cited. There is no requirement to cite these works unless the editor has indicated otherwise.

Answer：Thank you for your reminder. Regarding Item 9 (Reviewer-recommended literature), we have carefully reviewed all reviewer comments. We confirm that reviewers did not explicitly recommend or require us to cite any specific previously published works in their comments. Therefore, we will follow your guidance and refrain from making any mandatory additional citations.

**Response to reviewer**

**Referee #1**

| **Comment** | Response **and Change** |
| --- | --- |
| 1. The enhancements to ResNet50 (BN, PPL, hidden layers) are incremental. The manuscript should better articulate the novelty compared with prior deep learning approaches for UAV-based crop classification (e.g., DeepLab V3+, Bi-LSTM-Attention, MSNet). A clearer positioning of the contribution in relation to state-of-the-art models would strengthen the work. | We appreciate your valuable feedback. Your insightful and constructive point regarding the need for clearer articulation of this study's innovations compared to existing state-of-the-art deep learning models (such as DeepLab V3+, Bi-LSTM-Attention, and MSNet) is profoundly important, and during the model selection phase, we thoroughly considered these excellent architectures. However, as you are aware, high-resolution remote sensing images captured by UAVs exhibit significant spatial contextual features, and the optimal patch size is crucial for effectively capturing these characteristics. Our preliminary experiments and analysis indicate that the structural design of the encoder-decoder or sequence models imposes specific requirements or limitations on input dimensions, making it difficult to directly adapt to the input patch size (57×57) we determined through systematic experimentation. To determine the optimal input scale scientifically, we designed an image patch size screening experiment (detailed in Section 2.3.1 of the main text). Using the ResNet-50 architecture with full-band data as the input, we tested four patch sizes: 37×37, 47×47, 57×57, and 67×67. The experimental results (Section 3.1) consistently show that the 57×57 size achieves the highest classification accuracy on the validation set, as it strikes an optimal balance between pixel-level detail and plot-level contextual information. |
| 2.The ablation study indicates RGB+NIR+Edge as optimal, but the physiological/biophysical reasoning behind this combination is underexplored. Including vegetation index correlations (NDVI, CCI, etc.) or spectral signatures of maize and tobacco would justify why NIR and Edge bands are most discriminative. | We appreciate the reviewer's insightful suggestion. We agree that delving into the biophysical mechanisms underlying the optimal band combination is crucial for enhancing the value of this study. To this end, we conducted supplementary experiments to investigate the spectral characteristics of target crops. We collected 1,000 sample points for maize and tobacco and extracted their spectral reflectance values in the near-infrared (NIR) and red-edge bands. Additionally, we calculated NDVI to quantify the spectral differences across these critical spectral regions. The resulting cloud-rain map (now included in Figure 5 in the revised manuscript) clearly visualizes the distribution differences of these spectral features across the two crops. The analysis and discussion of these findings are presented in the section 3.2 |
| 3. Several equations (e.g., (1)–(4) for ResNet layer transformations, (5) for PPL, and (6)–(12) for BN propagation) are presented in a very generic form without proper derivation or sufficient explanation of the variables (e.g., fl,Wl,xl). To improve clarity, the authors should explicitly define all symbols at first use and connect the equations to their role in the modified ResNet50 architecture. 4. The mathematical formulations for BN and hidden layers (e.g., (6)–(14)) are largely restatements of standard definitions from deep learning literature. The manuscript would be stronger if these equations were contextualized to this specific application (UAV multispectral crop classification). For example, authors could explain how batch normalization interacts with multispectral input channels or why certain placements of BN (after convolution vs. hidden layer) theoretically affect convergence in their model. | We are grateful to the reviewers for their insightful comments on mathematical formulations. The manuscript has now been revised accordingly. We have specifically restructured the mathematical expressions for components, such as batch normalization and hidden layers, to closely align with their specific functions in the drone multispectral classification task. All symbols were explicitly defined, and their roles within the improved ResNet50 architecture in this study were clearly elucidated. See Sections 2.2.2, 2.2.3, and 2.2.4 for details. We believe that these revisions have significantly enhanced the clarity and depth of our methodology. |
| 5.The dataset is collected from a single location and two dates (Aug 19, 27, 2024). This may limit generalizability. The authors should discuss dataset diversity (seasonal variation, crop growth stages, environmental conditions) and whether the trained model would transfer to other geographies or times. | We sincerely appreciate the important comments from the reviewers. We acknowledge that datasets based on a single location and short time window may limit the generalizability of the model, which is a clear limitation of our study. This stems primarily from the realities of smallholder agriculture: crops are harvested shortly after data collection and field cropping patterns exhibit high interannual variability, making it extremely difficult to replicate data collection under identical conditions. We acknowledge this limitation and address it as a core focus of future research. We plan to systematically enhance the model's generalization and transferability by constructing multi-year, multi-season datasets and exploring advanced techniques, such as domain adaptation. |
| 6. While preprocessing steps (radiometric calibration, mosaicking) are described, details about class imbalance handling, labeler expertise, and annotation quality control are missing. Since mislabeling can strongly affect results, annotation reliability should be clarified.   9. Only overall accuracy, precision, recall, kappa, and F1 are reported. Given the class imbalance (tobacco vs. maize vs. background), class-wise metrics and ROC/PR curves would provide deeper insights into performance. | We sincerely appreciate the reviewers' valuable comments regarding data annotation quality and evaluation metrics. Regarding the class imbalance issue, we thoroughly considered and implemented targeted measures during both the research design and model-training phases. First, we strictly enforced balanced sampling (BalancedSubsetRandomSampler) to ensure identical sample counts for each class during training and validation (20,000 training samples and 5,000 validation samples per class), eliminating the impact of uneven class distribution at the data-input level. Second, we employed a Focal Loss function with optimized class weights ([0.4, 0.4, 0.2]). This adjusts the loss contribution from easy/difficult samples and different classes, further enhancing the focus of the model on the minority class. Regarding evaluation metrics, our current five-metric system (accuracy, precision, recall, Kappa coefficient, and F1 score) comprehensively reflects the model performance under strictly balanced data. Among these, the F1 score and Kappa coefficient are particularly sensitive and robust to class imbalances. Therefore, we conclude that this evaluation framework provides a sufficient and reliable measure of model performance under the current balanced dataset and a strictly balanced training framework. |
| 7. Results are compared only across enhanced ResNet50 variants. To demonstrate true advancement, comparisons with other architectures (e.g., EfficientNet, DenseNet, Vision Transformers) or traditional machine learning methods (e.g., SVM, RF with vegetation indices) should be included. | We sincerely appreciate the valuable suggestions of the reviewers. As you pointed out, comparison with a more diverse range of architectures is indeed crucial for demonstrating the state-of-the-art performance of the method. In response to your concern, we have systematically incorporated comparative analyses using multiple advanced models throughout our study. When selecting the models, we specifically considered the practical requirements of agricultural remote sensing applications. We focused on choosing classical and modern architectures comparable to ResNet-50, in terms of parameter count and computational complexity, as baseline models. These include ResNet-50, DenseNet-121, Darknet-53, and EfficientNet-B0, ensuring fairness and practicality in comparisons. These models offer a favorable balance between computational efficiency and performance, making them highly suitable for practical deployment on UAV remote-sensing platforms. We rigorously trained and evaluated all models on the same dataset, with detailed comparative results and in-depth analyses presented in the newly added Section 3.3. The results demonstrate that our improved ResNet-50 model maintains competitive advantages within this benchmark group, further validating the effectiveness and advanced nature of the proposed enhancement strategy. |
| 8. The discussion notes that PPL and hidden layers increased complexity without meaningful gains. A quantitative analysis of training time, parameter counts, FLOPs, or inference latency would make this trade-off clearer, especially since UAV systems often need efficient on-board or near-real-time processing. | We appreciate the reviewer's insightful comments. The considerations raised regarding the balance between model efficiency and performance are crucial for practical applications targeting drone platforms. In the revised manuscript, we have provided a more thorough discussion on this matter. As you observed, while the pyramid pooling module and additional hidden layers yield limited performance gains, our quantitative analysis confirms that the increases in parameters, computational load, and training time introduced by these components are negligible. This is because they constitute compact, efficient add-ons that do not impose substantial additional computational burden. To evaluate the comprehensive performance and stability of different model configurations more scientifically, we systematically supplemented Table 7 with the sample standard deviation as a key metric. This indicator effectively reflects the robustness of each model across multiple runs, which is crucial for assessing the reliability of a practical deployment. The analysis shows that the final model variant we adopted achieves an optimal balance between accuracy and stability, while maintaining essentially unchanged computational efficiency. We acknowledge that incorporating a more detailed real-time inference latency analysis will further strengthen future research. We have included this direction in our future research plans, and appreciate the reviewers' valuable perspectives on this matter. |
| 10. The study mentions overfitting with hidden layers, but no learning curves are provided. Including training/validation loss curves would help verify convergence behavior and overfitting tendencies. | We appreciate the reviewer’s suggestion. Your recommendation to validate the model convergence behavior and overfitting tendencies through the learning curves is crucial. We comprehensively documented and visualized the training processes of all models, incorporating the training and validation loss curves for eight distinct models as Figure 7 in the paper. These learning curves clearly reveal the convergence dynamics and generalization behavior of each model during training, providing direct evidence for the observed phenomenon of “hidden layers potentially causing overfitting.” Through an in-depth analysis of these curves, we further validated the convergence stability of the different model configurations and quantitatively compared their overfitting tendencies. A detailed analysis and discussion are presented in Section 3.4 of the paper, where these supplementary materials provide essential grounds for evaluating the stability of the model-training process. |
| 11. Page 13: The manuscript mentions 5-fold cross-validation but reports only mean values. Reporting standard deviations would better indicate robustness and variance across folds. | We sincerely appreciate the reviewer's important suggestion. We agree that reporting only the mean fails to adequately demonstrate the stability of the model's performance. To address your concern, we have systematically supplemented the standard deviation for each evaluation metric in Table 7, which clearly reflects the variability in model performance across the four random seeds. Additionally, to more intuitively demonstrate the distribution and dispersion of baseline model performance, we introduced Figure 6: Box Plot Comparing Baseline Model Performance. These additions provide stronger support for the robustness of our conclusions, enabling readers to comprehensively evaluate the consistency and reliability of the performance of each model. A detailed analysis and discussion of the relevant results can be found in Section 3.4. |

**Referee #2**

| **Comment** | Response **and Change** |
| --- | --- |
| The presented work in this manuscript is technically vigorous and well-structured, with strong experimental validation, the paper is suitable for publication. However, it is suggested for the authors to include a brief runtime/complexity analysis or at least a qualitative discussion of computational feasibility.. | We sincerely thank the reviewers for their recognition of this research and valuable suggestions. We have supplemented and refined the relevant analyses in the Discussion section of the paper according to your guidance. For details, please refer to the Discussion section of the paper. |

Once again, I extend my heartfelt gratitude to all the reviewers and editors for their valuable time and hard work. I wish you all good health and success in your endeavors! May your journal continue to thrive and grow in prestige!
